# A Narrative Review of the Digital Equity Gap of Apps for Cigarette Smoking Cessation for Persons Living in the Hispanosphere

**DOI:** 10.1007/s40429-024-00607-6

**Published:** 2024-11-06

**Authors:** Roger Vilardaga, Charlotte Stoute, Dana Rubenstein, Oluwatosin Akingbule, Madeline Gray

**Affiliations:** 1 Department of Implementation Science, Wake Forest School of Medicine, 525 Vine Street, Winston Salem, NC NC 27101, USA; 2 Psychiatry and Behavioral Sciences, Duke University School of Medicine, Durham, NC, USA

**Keywords:** Apps, App label, Smoking cessation, Digital equity, Hispanosphere, Anglosphere

## Abstract

**Purpose of Review:**

The Hispanosphere is a vast region of the world that has received little attention in the digital health literature. No study to date has examined the availability and quality of publicly available mobile applications (apps) for cigarette smoking cessation in this region. Three coders utilized the American Psychiatry Association (APA)’s Brief App Evaluation Model Screener (Brief-AEM Screener) to evaluate the quality of the label and public-facing screens of smoking cessation apps in Spanish. Availability of apps in the Hispanosphere was compared to availability of apps in the Anglosphere.

**Recent Findings:**

We identified and reviewed 19 apps in Spanish in Google Play. The median score using the Brief-AEM Screener was 63 out of 100 suggesting generally acceptable app quality and features according to the quality standards for digital health tools proposed by the APA. However, we found (1) notable inaccurate and misleading labelling claims, (2) poor grammar or incomplete translations, and (3) a lack of cultural and linguistic adaptation to countries in the Hispanosphere. Our comparison of smoking cessation apps between the Hispanosphere and the Anglosphere suggested that there is a large digital equity gap between these two regions, with a four to sevenfold gap in app availability.

**Summary:**

There is a relative shortage of quality and quantity of digital health apps for smoking cessation in the Hispanosphere. To ensure the cultural appropriateness of those digital interventions, it is essential that developers of digital health tools establish community partners in the region prior to developing apps for smoking cessation.

## Introduction

**Fig. 1:**
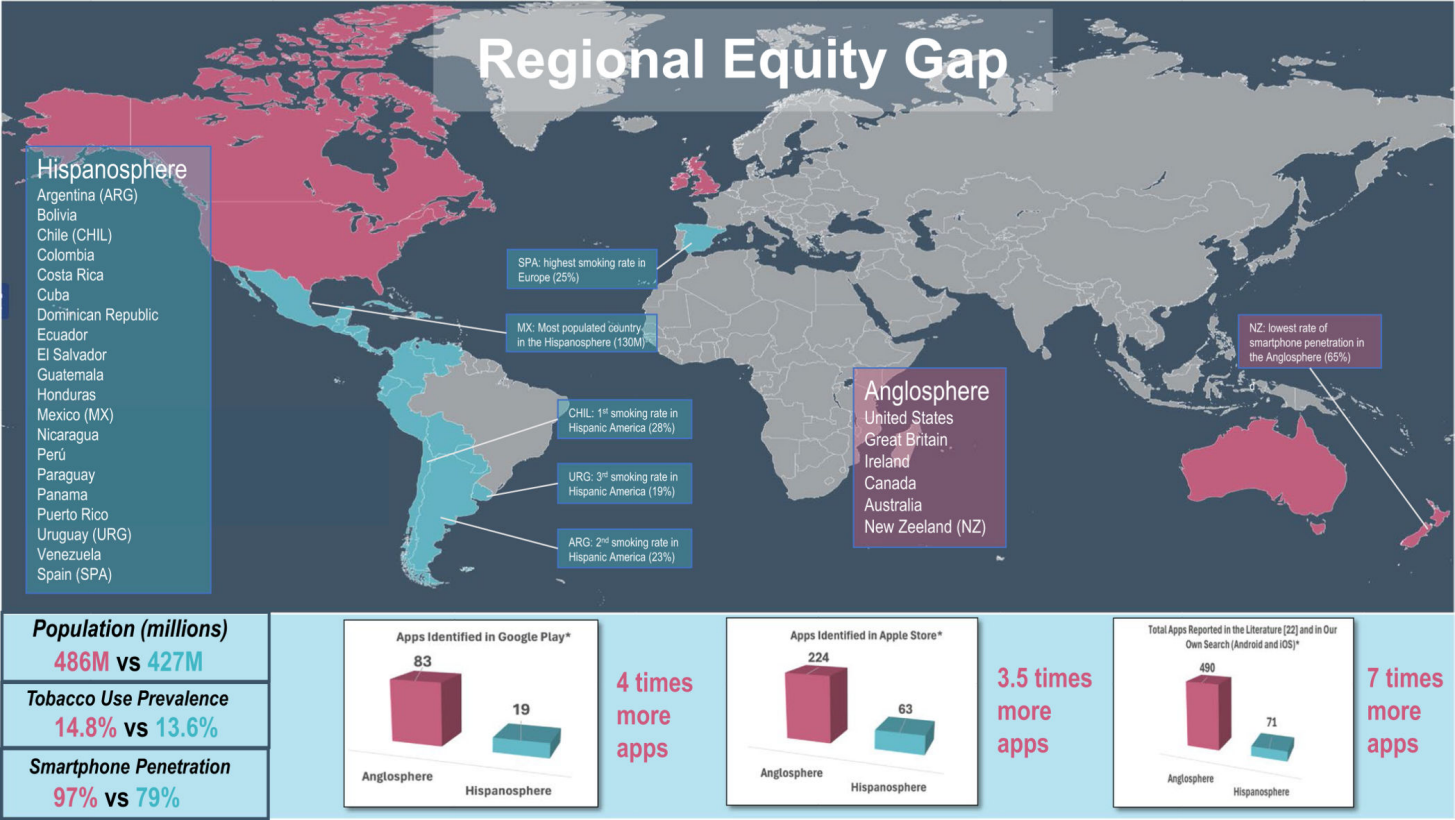
World map comparing global statistics in population, tobacco prevalence, smartphone use, and availability of Android apps between the Anglosphere and the Hispanosphere

In 2022, there were 496 million native Spanish speakers around the world, 596 million including all Spanish speakers regardless of their native status. This represents 7.5% of the world population, making Spanish the 2nd largest native language of the world, after Mandarin [1]. The growth of Spanish is projected to continue. The United States is estimated to be the second largest Spanish speaking country in the world by 2060, with 27.5% of its population of Hispanic origin. The term ´Hispanosphere´ refers to a vast geographical area in the world – which overlaps but is distinct from Latin America – where Spanish language serves as a ‘koine’ or common vessel that involves a shared sense of identity, cultural practices, and traditions. This vast region is mostly comprised of 20 countries distributed across the Americas and one in Europe (Spain). Hispanic America encompasses all of the Spanish-speaking countries of the Americas and is one of the areas of the world with the largest intraindividual racial diversity, with genetic ancestry from America [2, 3], Africa [4], Asia [2, 3], and Europe [4] (see Fig. 1 for a map and list of countries).

Cigarette smoking is a leading risk for early death, with global studies estimating that about 8 million people die prematurely from smoking each year [5]. Although smoking and smoking-related death rates have decreased in recent decades, about 15% of deaths globally are attributable to smoking [6], and 80% of people who use tobacco worldwide live in low and middle income countries [7]. Tobacco use remains high in Hispanic America [8], which experiences one of the highest levels of inequality in the world [8]. Countries including Uruguay, Argentina, Chile, Spain, and Cuba have high smoking and tobacco use rates, despite government and policy efforts that have helped reduce rates in recent years [6, 9].

Digital health technologies have become widely available worldwide. In 2018, it was estimated that more than 325,000 health apps had been developed, and that 1.7 billion people had downloaded mobile apps with a focus on health [10]. This widespread availability of health apps has the potential to impact the Hispanosphere [11, 12], with recent market data suggesting that mobile devices represent 80% of the market share in Latin America, and have remained consistently above the desktop and laptop computer market since 2020 [13, 14].

Despite this potential, little is known about the quality and availability of digital apps for smoking cessation in the Hispanosphere. Previous studies have reviewed the scientific literature to identify the clinical evidence of smoking cessation mobile health interventions in Latin America. These studies have focused on the design rigor and clinical evidence supporting the use of these interventions, two fundamental requirements for a health app that claims to treat or prevent disease [11, 12]. However, only a very small portion of all mobile apps available to the public that are branded useful for smoking cessation have been rigorously evaluated in clinical trials [15]. Nonetheless, the development and availability of smoking cessation apps continue to grow without regard to this important foundation. Therefore, from a public and global health standpoint, it is critical to evaluate the quality and availability of smoking cessation apps for Hispanic countries around the world.

The first goal of this review is to evaluate the quality of smoking cessation apps that are available in Spanish in the Hispanosphere in a widely available software platform (i.e. Google Play) using a simple, comprehensive, and theoretically grounded digital health evaluation system. The second goal of this review is to compare the quality and availability of smoking cessation apps in the Hispanosphere to the availability of smoking cessation apps in a selected group of high-income countries also known as the Anglosphere. The Anglosphere is a group of English-speaking high-income countries with close political, military, and cultural ties [16]. This region, distributed across America, Europe, and the Western Pacific, includes New Zealand, Australia, United Kingdom, Ireland, Canada, and the United States (see Fig. 1). This regional equity comparison allows us to examine the need for large-scale smoking cessation interventions in the Hispanosphere relative to an economic and politically privileged area of the world. To cast our comparison, we will examine most recent data on prevalence rates of smoking in each of the countries that comprise those regions. Overall, the goal of this narrative review is to illuminate specific inequities in the availability and quality of digital interventions for smoking cessation in these two key regions of the world.

## Methods

**Fig. 2:**
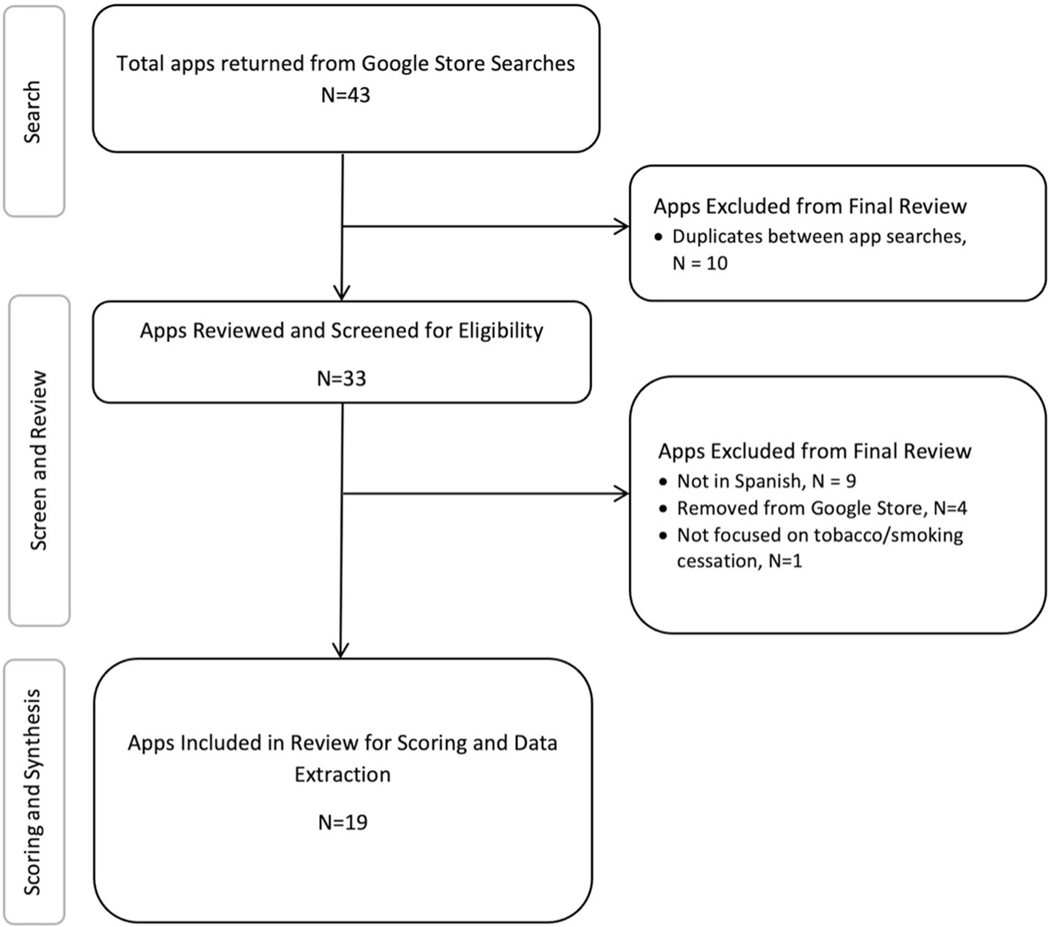
Flow Diagram of returned apps for smoking cessation from Google Play (Android) from search conducted in October 2023, followed by screening, review, ratings, and data extraction processes occurring between October 2023-May 2024, synthesis completed by May 2024

A search was conducted in Google Play to extract a list of smoking cessation apps in Spanish language on October 22, 2023 (see flow diagram in Fig. 2). According to a 2024 market study, Android devices represent 83.2% of all the smartphone market in Latin America, with Apple iOS devices at 16.5% [17]. With a focus on this market, our review of Android apps addressed the needs of the majority of users of apps for smoking cessation in that region. The search tool in Google Play was used to extract the list of apps. We used the following key terms to conduct this search: *dejar de fumar* [*quit smoking*], *apps para dejar de fumar* [*apps to quit smoking*], and *tabaco* [*tobacco*]. This procedure selects apps in a way that would reflect a layperson’s behavior and has been used in prior research [15]. We used an ‘incognito’ window in Google Chrome and Firefox to perform our search and prevent the Google Play from generating results in English (see Supplement 1).

Results were screened to remove app duplicates, apps that appeared not to be in Spanish (i.e., per the language used in Google Play’s screenshots), or apps that did not have smoking cessation as the primary goal (e.g., substance use cravings, apps targeting both alcohol and tobacco use). From each result, raters (CS, DR) reviewed the app quality using the Brief Version of the American Psychiatric Association (APA)’s App Evaluation Model Screener (Brief-AEM Screener [10, 18]; see Supplement 2). We also extracted from Google Play (1) what could be described as the “app label”, that is, a description of the app’s purpose, active ingredients, and expected benefits; (2) the country of origin of the app developer; (3) the privacy policy; (4) the number of app installations; (5) the average star rating; (6) the number of reviews; (7) the price, and (8) the date of last version update.

The APA’s AEM Screener was originally developed to address the need for a simple and comprehensive framework to critically evaluate the quality of the large amount of smartphone applications for mental health conditions that have proliferated in the last decades [10, 18]. APA’s AEM Screener was developed based on a systematic framework that included five foundational levels of evaluation: background information, privacy and security, evidence-base, ease of use, and data integration [10]. In this review we used the abbreviated version of the AEM Screener, the Brief-AEM Screener, which includes eight essential questions that address each of the five levels of evaluation [19]. Two reviewers fluent in Spanish (CS and DR) independently reviewed the final list of apps and coded each of the apps based on the Brief-AEM Screener. A ninth component of the quality assessment (not used for calculating the quality score) was quality of language translation, which was used to assess whether the content was correct, appropriate, and completely in Spanish. These results are summarized qualitatively for the purpose of this review. Evaluation of these quality criteria was solely based on the available app ‘label’, that is, all the information publicly reported by the developer about the purpose, functionality, clinical benefit, and safety of an app prior to installing this software in a personal device.

After reviewing and rating each of the apps independently, the team met to review findings, compare ratings, and discuss inter-rater discrepancies, which were resolved through consensus and final assessment by RV (first author, also fluent in Spanish). The criteria for app ratings using the Brief-AEM Screener (provided in Supplement 2) was utilized to systematically rate all the apps, which were confirmed by all raters prior to scoring. Each of the criteria scores were summed, divided by 8 and multiplied by 100 to create a 0–100 rating score, with 100 indicating the highest comprehensive level of app quality, and 0 the lowest. Similar review procedures using both the Brief-AEM and the AEM Screener have been employed in the past [15, 20].

The final step was to estimate the number of apps for smoking cessation available to the public in the Anglosphere, and the number of apps for smoking cessation available to the public in the Hispanosphere in the Apple Store. We utilized two strategies to extract this data. The first and most ecologically valid method was to extract a count of the number of apps available in each of the corresponding app stores. This method directly resembles what a user who wants to quit smoking might be able to do to identify an app for smoking cessation. Since language is a fundamental determinant of app accessibility (i.e., the actual ability to read, comprehend, and benefit from a digital product) for people living in Anglo countries, we conducted a direct search in Google Play and the Apple Store in May 2024 with the same key terms used for our previous search in English (e.g., “quit smoking”). The same search was conducted in Spanish in the Apple Store. We excluded apps that a) were duplicates, b) did not have a focus on smoking cessation, and c) provided app images (i.e., screenshots) that were not in English. All remaining apps were tallied and retained in our total count. Finally, the second strategy we employed was to consult the peer-review literature to identify the number of apps for smoking cessation reported in both English and Spanish. Therefore, we examined the scientific literature to extract any reports or counts about the number of apps for smoking cessation available in English and Spanish.

### Summary of Findings

Forty-three apps were initially identified by the team based on Google Play searches for smoking cessation apps in Spanish. Among those, 10 apps were duplicates, and nine apps had the app label in Spanish but app images in English, which suggested that the apps had not been fully translated and were therefore excluded. Four apps stopped being available in Google Play during our period of analysis between October 2023 to May 2024, therefore those apps were excluded as well. Finally, one app was excluded because it did not have a targeted focus on tobacco/smoking cessation (i.e., it targeted both tobacco and alcohol). This rendered a total of 19 apps for smoking cessation to be reviewed with the Brief-AEM Screener. A flow diagram (Fig. 2) reflects the search and review process.

### Overview of App Development, Cost, and Popularity

Among the 19 apps in Spanish that we identified, 6 were developed in Spain (31.5%), 2 in the United Kingdom (10.5%), 2 in France (10.5%), and 2 in Brazil (10.5%). The remaining apps were developed in Mexico, Russia, Italy, Germany, the Czech Republic, Cyprus, and Ukraine, with one app (less than 5%) for each country. All the apps were free except for *QuitNow PRO: Dejar de Fumar,* an advanced version of the free app *QuitNow: Dejar de Fumar,* that had a cost of $25.99.

Four apps did not have a star rating. Among the remaining apps, the star rating ranged between 4.2 and 5 (Mean = 4.64; Median = 5). Among the apps that were rated, the number of reviews ranged between 30 and 158,000, with a median of 1,400 (Mean = 23,197). The most downloaded app had 5 million downloads, and the least downloaded app had 1,000 downloads, with an average above half million (Mean = 542,421; Median = 50,000; see Table 1).

### Overall Quality Assessment

The results of quality assessment with the Brief-AEM Screener are as follows. The identified apps had a median quality score of 63 (on a 0–100 scale). Over one third (36.8%) of the apps had a score of 75, and 52.6% of the apps had a score above 50. The remaining apps had either a score of 50 (21%), or a score below 38 (26.3%; see Table 1 for more details). The criterion that was met most frequently was “ease of use” (Criterion G), which was met by 100% of the apps. This was followed by having a clinical foundation (Criterion F; 79%), being updated in the last 180 days (Criterion B; 74%), and collecting, using, and transmitting sensitive data securely (Criterion D; 68%). The remaining criteria were met by a smaller proportion of the apps such as working in both Android and iOS (Criterion A; 58%), collecting data that could be easily shared and interpreted in a way that is consistent with the stated purpose of the app (Criterion H; 32%), having a transparent and clear privacy policy (Criterion D; 21%), and evidence of specific benefit from a research or academic institution (Criterion E; 5%).

### App Labelling and Images

Our qualitative review of app labels and images in Google Play in conjunction with the Brief-AEM Screener, led to the following themes, which are presented below with some specific examples.

#### Acceptable Evidence‑based Content

According to the app labels, one app, *Dejar de Fumar Ya – Smoke Free* had been tested in large clinical trials, therefore meeting one of the most rigorous criteria: Criterion E for clinical evidence. In addition, 15 apps appeared to have a clinical recovery foundation (Criterion F), and 12 apps described in their label what could be characterized as content based on best-practice smoking cessation guidelines (e.g., US Clinical Practice Guidelines or World Health Organization) [e.g., 21]. These strategies generally included psychoeducation, the use of distraction, goal setting, anxiety management, crisis support, social support, inspiration, and rewards. A very common feature was the inclusion of cigarette trackers, money savings, and different types of graphic displays to visualize this data. Other apps also included features such as a diet, exercise, and a sleep program (i.e., *CigArrete*). Finally, *Stop Tabaco, Dejar de Fumar*, had an excellent design and evidence-based content, however, it lacked any privacy policy information in Google Play (see below for a description of other apps that did not meet this standard), and the app had not been updated since June 2022.

#### Quality of Spanish Language and Translation Completeness

Only 21% of all the reviewed apps had a privacy policy that was clear and accessible before use (Criterion C). Failure to meet this criterion occurred in 10 out of 15 cases due to including a privacy policy statement that was not written in Spanish. Only three developers had their privacy policy in Spanish, one from Mexico (i.e., *Dejar de Fumar – Alive*), one from the Czech Republic (i.e., *Adiquit: Deja de Fumar*), and one from Spain (i.e., *RespirApp – Dejar de Fumar*). The remaining developers had privacy policies in English, with only two exceptions, one in Russian (i.e., *SWay: Dejar/Menos de Fumar*) and one in Italian (i.e., *Fumar menos, Smoking less*). Only one app included a privacy policy in multiple languages (i.e., *Dejar de Fumar, Alive;* four languages). Surprisingly, among the six apps developed in Spain, five had their privacy policy written in English.

Four apps reviewed in this study had an app label in Spanish that had grammatical errors, misleading statements, and/or non-sensical sentence content or structure, possibly poor/mistranslations. The app *DWS: Mostrador sin Humo*, included the following motivational/feedback statement in response to someone who had quit smoking for two days: “Miles de personas dejan de fumar al día, muriendo por ello. Simplemente deseo que no seas uno de ellos” [“Thousands of people quit smoking everyday, dying as a result of it. I simply wish that you are not one of them”]. The app *Sway: Dejar/Menos de fumar*, has a name in Spanish that lacks a clear meaning (i.e., “menos de fumar” [less of smoking]). *Qwit (Para Dejar de Fumar),* and *Dejar de Fumar – CigArrete,* included significant grammar errors in the app label and app screenshots, suggesting that the language had not been reviewed by a native speaker. Most notably, the app *Dejar de Fumar – Quit Smoking,* appeared to be written by a software translation tool, and it included a long string of non-sensical sentences (i.e., “Dejar la nicotina la vida se vuelve más plena, mejora la salud y aumenta el bienestar, por lo que el humo antes de que sea demasiado tarde y cuando rompemos el hábito juntos, se hace mucho más fácil” [“Quitting nicotine life becomes more full, health improves and wellbeing is augmented, through which the smoke before it is too late and when we break the habit together, it becomes much easier”]). Further, one app had a statement that could be harmful: “Recuerda que cada día que te dices a ti mismo que lo fumes te estás acercando a tu objetivo…” [“Don’t forget that everyday that you tell yourself to smoke it you are getting closer to your goal…”].

#### Few Apps Developed in the Hispanosphere

As noted earlier, only 26% of the apps were developed within the Hispanosphere, with a concentration in Spain, a high-income country. Only one app was developed in Mexico, and none in other countries of the Hispanosphere. The wide range of countries involved in the development of smoking cessation apps available to the Hispanosphere was substantial, with a concentration in Europe (Czech Republic, Cyprus, France, Germany, Italy, Ukraine, United Kingdom, Russia) and Luso America (Brazil). The lack of an app developed in the United States was notable given its large Hispanic population (i.e., ~ 20%). It was also observed that none of these apps did appear to be culturally adapted to the cultural and living conditions of Hispanic America. For example, 14 apps indicated to have a money saving feature. However, the only two available currencies to track money savings in all the apps reviewed were Euros (9 apps), Dollars (4 apps), and Pounds (1 app).

#### False Claims or Misleading Information

The app label for *Adiquit: Deja de Fumar*, made false claims about the scientific significance of their intervention. The label stated that “Adiquit es la única aplicación basada en el conocimiento científico y la práctica clínica real.” [“Adiquit is the only application based on scientific knowledge and real clinical practice”]. The label further states that “Adiquit es la ayuda más eficiente disponible en el mercado…” [“Adiquit is the most efficient help in the market”]. None of these statements are accurate based on current evidence. Other studies have demonstrated the efficacy of smoking cessation apps [22], including one of the apps reviewed in the current paper, *Dejar de Fumar Ya – Smoke Free* [23].

The developers further stated that “los estudios clínicos muestran que las posibilidades de dejar de fumar con éxito con Adiquit son hasta seis veces mayores en comparación con dejar de fumar sin ayuda profesional” [“clinical studies show that the odds of quitting smoking with Adiquit are up to six times higher compared to quitting smoking without professional help”]. In our review of the *Adiquit*’s website, the developers indicated that the app was based on several large clinical trials that pioneered the efficacy of a web-based intervention that preceded the *Adiquit* app [24–26]. They also reported a pilot study (N = 34) [27] that had user acceptance and a technical assessment of the app as the primary outcome. The evidence provided by those references is sound. However, the prior claim that *Adiquit* was the only app based on scientific knowledge seemed an overstatement.

Finally, we found another app, *Dejar de Fumar – Quit Smoking,* that made a statement in the app label that “nicotine has been recognized for a long time as one of the most dangerous habits leading to serious health conditions.” While statements of this kind are not directly harmful, they can contribute to the misconception that nicotine is the only harmful chemical packaged in combustible tobacco products such as cigarettes. This type of statement could deter the public from utilizing or adhering to Nicotine Replacement Therapy products, which is the most cost-effective, accessible, and widely available pharmacotherapy for smoking cessation.

### Regional Equity Comparison Between the Hispanosphere and the Anglosphere

**Fig. 3:**
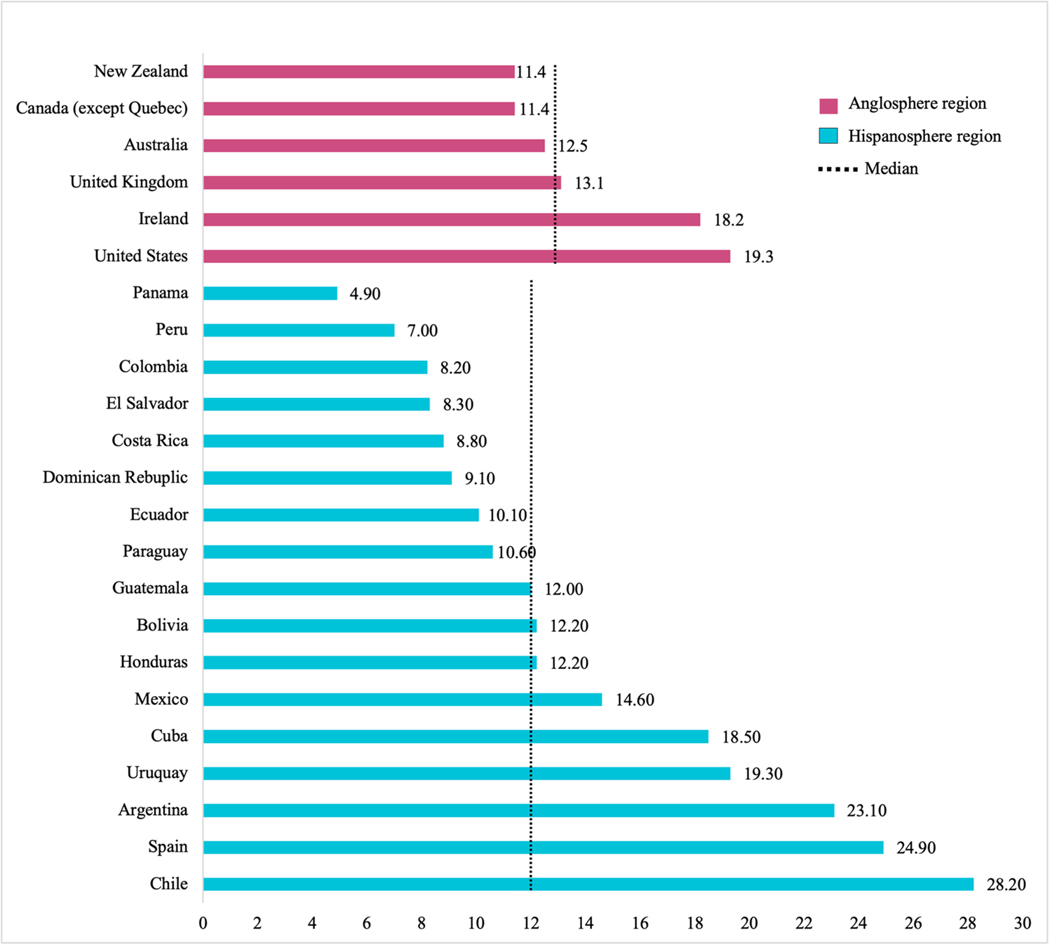
Available Prevalence of Tobacco Smoking by Country in Anglosphere and Hispanosphere Regions of the World in 2024 (%). Median Anglosphere = 12; Median Hispanosphere = 12.8.*. *Source: World Health Organization. WHO global report: mortality attributable to tobacco. 2024

Figures 1 and 3 show the results of a comparison of the need and access to digital health apps between the Hispanosphere and the Anglosphere. Figure 3 shows the prevalence of tobacco smoking in each of the target countries highlighted by region based on the most recent World Health Organization global report on trends in tobacco use [28]. The 17 countries included in the Hispanosphere region for which data were available had an average tobacco smoking prevalence rate of 13.65%, and a median of 12%. There was a wide range in the smoking rates across countries in this region, from 4.9% in Panama to 28.2% in Chile. On the other hand, countries in the Anglosphere region had an average prevalence rate of 14.32%, with a median of 12.8% and a narrower range from 11.4% in Canada to 19.3% in the United States.

Prevalence of tobacco smoking was equal or higher than the median of 12% in 8 out of the 17 countries with available data in the Hispanosphere region (24%, 14.6%, 12%, 18%, 23%, 28%, 12%, 19%). Conversely, prevalence was higher than the median of 12.8% in 3 out of 6 countries in the Anglosphere region (19%, 18% and 13%). Spain, the only country in Europe of the Hispanosphere, had one of the highest prevalence of tobacco smoking (25%) for countries in Europe.

The population size of these two regions is similar, with 427 million in the Hispanosphere region versus 486 million in the Anglosphere region (Fig. 1). Access to mobile technology differed across the two regions. Smartphone adoption in the Hispanosphere (including all Latin America) was estimated to be 79% in 2022, and it is projected that it will reach 93% in 2030 [13]. The forecast is that monthly data traffic in this region will increase by 350% in the next five years. In fact, Hispanics have been categorized by marketing reports as ‘super mobile consumers’ [29]. Conversely, smartphone adoption was markedly higher across the different countries in the Anglosphere. The median percent of smartphone penetration in this region in 2024 is 97%, with New Zealand having the lowest of all (65%). Smartphone penetration exceeded 97% in the remaining countries of that region.

Finally, availability of apps for smoking cessation across these two regions varied substantially depending on the search method employed. We used a direct app store search method, more ecologically valid and consistent with the purpose of this review, and a second method that relied on the peer- review literature. As noted earlier, using our first method, our search of apps for smoking cessation in Spanish in Google Play initially identified 19 apps. When performing the same search in English, we identified a total of 83 apps. In the Apple Store we identified 72 apps for smoking cessation in Spanish and 224 apps for smoking cessation in English. The results of our second method was an independent market analysis performed by SensorTower.com in 2020, that indicated that on April 2020, there were 490 apps for smoking cessation in English language [22]. In contrast, we found a meta-analysis of the mHealth literature in Latin America that found only seven mobile interventions being tested in both Luso (Brazil) and Hispanic America (Mexico, Peru). Among them, only one could be characterized as a digital health app (most of the mobile interventions had a telephone or web-based component). Figure 1 provides a summary of all these findings, by presenting key similarities and differences in population, smartphone access, and availability of smoking cessation apps across these two geographical regions.

### Discussion of Findings

In this narrative review of smoking cessation apps available across the Hispanosphere, we found that the majority of these apps had high levels of ease of use, an appropriate clinical foundation, appropriate security and privacy features, and had been recently updated. While the quality and content of the app label of most of the apps were acceptable, the score of some apps on the Brief-AEM Screener was relatively lower due to technical and app management factors unrelated to the app content and design. In addition, the privacy features for the majority of these apps were not clear or accessible for the target audience (i.e., were not in Spanish), most of them had not been directly tested in a clinical trial, and very few allowed sharing data with others for a clinical purpose. The median score on the Brief-AEM Screener was 63 (possible range = 0–100), suggesting that the quality of these apps was generally acceptable according to the proposed five levels of compliance put forward by the American Psychiatric Association for digital health apps: 1) appropriate background information, 2) privacy and security, 3) evidence-base, 4) ease of use, and 5) data integration.

This review also shed light about other aspects relevant to the development of apps for smoking cessation for people living in this region. Specifically, most developers did not appear to consider the importance of translating all the public-facing elements of the application, such as having a privacy policy that was in Spanish or having the app label reviewed by a native Spanish speaker. Likewise, country-specific aspects of the apps such as including a menu of currencies available in all potential countries were also missing, and thus app developers did not account for the broad range of countries in which those apps could be utilized other than those in the European Union, England, and the United States. We did not review the cultural appropriateness of the app content and design, but it was notable that out of all the apps reviewed, the majority were developed in northern European countries with a very different cultural background than Hispanic countries. Furthermore, only seven were developed in the Hispanosphere, and six of those in a high-income country (Spain), which could have also limited the adoption and generalizability potential of these apps to other countries within the same region. Two were developed in Luso America (Brazil), but this Latin American region has a different cultural, historical, and linguistic background than the Hispanosphere, which does not fully resolve the cultural appropriateness of the apps.

Note that it is possible that many of these apps were not developed with the intention to be used globally. However, we did not find this intention directly communicated in any of the app labels (e.g., a statement about the target country or region of the Hispanosphere). Prior literature has emphasized the need to develop digital interventions that are culturally and linguistically tailored to different Hispanic regions [11, 12], and several studies have culturally adapted mobile interventions for specific regions of the Hispanosphere [30–33]. The use of known methodologies during the design of these apps, such as community participatory methods and/or user-centered design would ensure the cultural and regional input of the target audience [34, 35]. Formally, this could include establishing community advisory boards with members of the target audience, or conducting one on one community consultations with key informants of a broad range of Hispanic countries. Informally, this could be approached by hiring into the company’s app development team employees that are bilingual and/or reside in those countries.

False or inappropriate label claims were rare among the apps, but there were some notable exceptions, which highlights the overall lack of oversight by Google Play for the accuracy of marketing claims and health statements made by these companies. In terms of financial barriers, all apps were free except for one (which also had a free version). This should contribute to the wide-scale adoption and dissemination of these apps. In fact, these apps, on average, had over half a million downloads. However, the privacy policies of many of these apps clearly stated that personal data could be shared with third party vendors, which from a health equity standpoint calls to question the ‘real price’ of these apps [36]. Overall, these findings are consistent with a recent review that used the AEM Screener to evaluate 228 smoking cessation apps for an Anglo audience [15]. While the cited review used the full AEM Screener and did not compute an overall score of app quality based on the Brief-AEM Screener version, there were some notable similarities in the findings. For example the authors found a lack of availability of privacy policies in 25% of the apps, and only 6% of apps had published studies supporting their efficacy [15]. Similarly, in our study, 21% of the apps had a clear privacy policy, and 5.2% of the apps had a published study supporting their evidence.

Our review also shed light on the digital equity gap of apps available for persons living in the Hispanosphere versus the Anglosphere. While the populations of these regions of the world are very similar in size, and have similar rates of smoking and smartphone penetration, there is a significant inequity in the amount of available digital interventions for smoking cessation across those regions. Our review estimated that there was a four to sevenfold gap in available digital tools for smoking cessation between the Anglosphere and the Hispanosphere. Discussing the socio-economic and political reasons for the lack of development of digital health tools for smoking cessation in this region goes beyond the scope of this paper. However, it takes a particular governmental, technology, healthcare, and entrepreneurial environment to promote the development of digital health interventions and their sustainment over time. Even in an ideal environment, entrepreneurship is a challenging enterprise that is subject to venture capital trends, regulatory challenges, shifts in funding priorities, and the appropriate use of subject matter expertise.

This study had several limitations. First, our review of apps available in Spanish in Android did not take into account the estimated 16% of persons living in the Hispanosphere that are iOS users [17]. Second, our focus on the Hispanosphere excluded other Latin American countries, such as Brazil and French Guiana, and included Spain. However, Hispanic countries share an essential element, which is the use of Spanish language as a koine or vessel for the transmission of a set of shared values and cultural practices across Spain and Hispanic America up until today. The absence of this koine limits the interoperability of apps in Portuguese and French with Hispanic persons in the region. Third, our study did not conduct an in-depth review of each of the apps for smoking cessation available in the Hispanosphere. Downloading each of the apps and testing them for a limited period would have provided more extensive knowledge about the characteristics of those digital health tools. However, our intent in this review was to evaluate public-facing information available to anyone in the Hispanosphere *prior* to deciding to install an app on their personal device. Thus, the value of this review resides on how claims about the purpose, functionality, clinical benefit, and safety of an app are accurately and faithfully presented to the public. Forth, as noted by other authors, the availability of health apps in the App Stores is subject to high volatility [15, 37]. During our review period, several apps ceased to exist in Google Play, and in a prior review [15] using the APA’s AEM Screener, the authors noted a large variation from year to year about the availability of apps in English in the Apple and Google Play Stores. In that context, our study represented a snapshot of currently available apps in Spanish in a highly fickle and fluid market during a specific time period. Finally, our review only focused on cigarette smoking cessation apps, excluding digital products focusing on vaping-only or cessation of other tobacco/nicotine products. While this focus on cigarette smoking limits the conclusions of this review to this type of tobacco product only, cigarette smoking represents the larger proportion of tobacco use in low and middle income countries [7].

## Conclusions

Apps for smoking cessation play a critical role in increasing access to evidence-based interventions that could mitigate the tobacco pandemic around the world. These digital tools can provide tailored, reliable, and affordable treatments at scale. The Hispanosphere is a vast region of the world sharing a common language and cultural traits that has received little attention in the digital health literature. In this review of smoking cessation apps available in this region, we found that while the evidence-based content of available apps for smoking cessation were generally acceptable, Spanish translations were incomplete or inadequate, and some developers made notable misleading or inaccurate claims in the app label. A comparison of need and access to smoking cessation apps between the Hispanosphere and the Anglosphere suggested that there is a large digital equity gap between these two regions, that is, the volume of digital tools available for this region is very small relative to their need. Finally, this study suggests that it is essential that digital health developers of smoking cessation apps establish community partners in the Hispanosphere prior to developing smoking cessation interventions for persons living in that region.

## Supplementary Material

Review Procedures: Search Strategy and Data Collection

American Psychiatric Association's Brief App Evaluation Screener (Adapted)

## Figures and Tables

**Table 1:** Smoking cessation-related apps on Google Play (N = 19) reviewed with the Brief-AEM Screener

App	Country of Origin	Number of Installs	Star Rating	Number of Reviews	A	B	C	D	E	F	G	H	AEM Screener
QuitNow: dejar de fumar	Spain	1,000,000	4.6	64,400	1	1	0	1	0	1	1	1	75
Kwit—¡Dejar de fumar!	France	500,000	4.2	11,500	1	1	0	1	0	1	1	1	75
Dejar de Fumar—Alive	Mexico	5,000	4.5	322	1	1	1	1	0	1	1	0	75
Adiquit: Deja de fumar	Czech Republic	10,000	NA	0	1	0	1	1	0	1	1	1	75
RespirApp—Dejar de fumar	Spain	10,000	NA	0	1	1	1	1	0	1	1	0	75
Qwit (para dejar de fumar)	Spain	500,000	4.6	10,800	1	0	1	1	0	1	1	1	75
Dejar de Fumar Ya—Smoke Free	United Kingdom	1,000,000	4.7	57,000	1	1	0	1	1	1	1	0	75
Dejar de fumar—CigArrête	France	100,000	4.6	1,400	1	1	0	0	0	1	1	1	63
QuitNow PRO: Dejar de fumar	Spain	50,000	4.9	6,630	1	1	0	1	0	1	1	0	63
Dejar de fumar—EasyQuit	Germany	1,000,000	4.8	103,000	1	1	0	1	0	1	1	0	63
Flamy—Dejar de fumar	Cyprus	500,000	4.5	16,500	0	1	0	1	0	1	1	0	50
Dejar de Fumar Asistente	Ukraine	5,000,000	4.8	158,000	0	1	0	1	0	1	1	0	50
Dejar de Fumar Ya—Asistente	Spain	10,000	4.8	534	0	1	0	1	0	1	1	0	50
Dejar de Fumar—ISMOKEY	Brazil	10,000	NA	0	1	1	0	0	0	1	1	0	50
SWay: Dejar/menos de fumar	Russia	50,000	4.6	1,270	0	1	0	0	0	0	1	1	38
Dejar de Fumar – Quit Smoking	United Kingdom	1,000	5	30	0	1	0	0	0	0	1	0	25
Stop Tabaco. Dejar de fumar	Spain	500,000	4.2	8,720	0	0	0	0	0	1	1	0	25
Fumar menos, Smoking Less	Italy	10,000	NA	0	0	0	0	1	0	0	1	0	25
DWS: Mostrador sin humo	Brazil	50,000	4.8	631	0	0	0	0	0	0	1	0	13
Statistic^2^		M = 542,421 Md = 50,000	M = 4.64 Md = 5	M = 23,197 Md = 1,400	58%	74%	21%	68%	5%	79%	100%	32%	M = 54.6 Md = 63

Criterion A: *“Does it work on both iOS and Android smartphones?*”; Criterion B: “*Has the app BEEN updated in the last 180 days?*”; Criterion C: “*Is there a transparent privacy policy that is clear and accessible before use?*”; Criterion D: “*Does the app collect, use, and/or transmit sensitive DATA? If yes, does it claim to do so securely?*”; Criterion E: “*Is there EVIDENCE of specific benefit from academic institutions, end user feedback, or research studies?*”; Criterion F: “*Does the app have a clinical/recovery foundation relevant to your intended use?*”; Criterion G: “*Does the app seem easy to use?*”; Criterion H: “*Can data be easily SHARED and interpreted in a way that’s consistent with the stated purpose of the app?*”; NA: Not Available; 2: M = Mean, Md = Median. The percentage represents apps classified as ‘Yes’ on each criterion relative to the number of apps included

## Data Availability

No human subjects datasets were generated or analysed during the current study.
