# Supplementary material for: A Narrative Review of the Digital Equity Gap of Apps for Cigarette Smoking Cessation for Persons Living in the Hispanosphere
Source: Curr Addict Rep. Author manuscript; Available in PMC 2025 Dec 1. (PMC12107453; doi:10.1007/s40429-024-00607-6)
Supplement: Review Procedures: Search Strategy and Data Collection [file NIHMS2054582-supplement-Review_Procedures__Search_Strategy_and_Data_Collection.docx]

# Supplement 1: Review Procedures: Search Strategy and Data Collection

## Search Strategy

1. Access: Use Google Store via desktop (<https://store.google.com/>).
   1. Specifications: Firefox internet browser (incognito mode, set to Spanish) to return apps in Spanish that are not influenced by device settings/search history. Google Chrome used to validate and search during review and reconciliation process.
2. Search Parameters:
   1. Terms entered:
      1. Search# 1= [“apps” + “para”+“dejar” + “de”+ “fumar”]
      2. Search# 2=[“dejar” + “de” + “fumar” + “apps”]
   2. Direct links to searches:
      1. Search# 1= <https://play.google.com/store/search?q=apps%20para%20dejar%20de%20fumar&c=apps>
      2. Search# 2= <https://play.google.com/store/search?q=dejar%20de%20fumar&c=apps>
   3. Date of initial searches: October 22, 2023
   4. List compiled with duplicates removed (23 apps total derived from search #1, and 33 from search #2; Apps 1-23 from search 1 duplicated in search #2, rendering 33 unique apps total)
3. Quality Control:
   1. Rating criteria operationalized in App Review Screening Tool and Ratings Criteria for author and rater use in review process (see Supplement 2).
   2. VPN verification: RV verified results using VPN for two selected countries (i.e., Mexico and Argentina) on May 16, 2024 to explore that searches performed were representative of results in two key geographic locations.

## Data Collection (Review and Extraction)

1. App Label reviewed and information ascertained and tabulated/recorded as follows:
   1. Information related to screener ratings are ascertained independently by raters and added into individual excel sheets, noting Y, N, or N/A as applicable for each individual app; reviewers also include notes about ratings and exclusion rationale as applicable.
   2. After individual ratings are completed and sent to main author (RV) these are merged and compared against each other to identify any discrepancies.
   3. All discrepancies identified are discussed by consensus and final decisions made by RV based on reconciliation meeting discussions and direct verification/review of app label. Final list of eligible apps included in data extraction and synthesis.
2. Data Extraction: For each app listed, all the following data are ascertained (variables/fields with * are data extracted and tabulated for synthesis and presentation)
   1. Country of Origin of App Developer*
   2. Number of App Installations*
   3. Star Ratings*
   4. Number of Reviews*
   5. App Label
   6. Privacy Policy (if available)
   7. App images
