# Supplementary material for: A Narrative Review of the Digital Equity Gap of Apps for Cigarette Smoking Cessation for Persons Living in the Hispanosphere
Source: Curr Addict Rep. Author manuscript; Available in PMC 2025 Dec 1. (PMC12107453; doi:10.1007/s40429-024-00607-6)
Supplement: American Psychiatric Association's Brief App Evaluation Screener (Adapted) [file NIHMS2054582-supplement-2.docx]

# Supplement 2: American Psychiatric Association’s Brief App Evaluation Model Screener (Adapted)

| CRITERION | STAGE | Evaluation Model Screener | Operationalization for Rating Decisions |
| --- | --- | --- | --- |
| A^1^ | Access & Background  Access & Background | 1. On which platforms/operating systems does the app work? Does it also work on a desktop computer?  [Adapted Item: *Does it work on both iOS and Android smartphones?*] | The item was adapted to allow a Y/N question and limit the number of platforms to the most widely available platforms in the world, iOS and Android. |
| B |  | 2. Has the app been updated in the last 180 days? | Evaluation of this item was limited to the review period. |
| C | Privacy & Security  Privacy & Security | 3. Is there a transparent privacy policy that is clear and accessible before use? | Any policies not in Spanish were considered neither clear nor transparent. |
| D |  | 4. Does the app collect, use, and/or transmit sensitive data? If yes, does it claim to do so securely? | The following procedures were used to code this criterion: (a) Apps that did not collect any sensitive data were coded as Y; (b) Apps that collected sensitive data and described technical, physical, or administrative processes to keep them secure were coded as Y; (c) Apps that collected sensitive data and did not describe technical, physical, or administrative processes to keep them secure were coded as N; (d) Apps that made contradicting statements in the privacy and security label were coded as N (e.g., claimed not to collect data AND transmit data securely); (e) Apps that had no privacy policy were coded as N. |
| E | Clinical Foundation  Clinical Foundation | 5. Is there evidence of specific benefit from academic institutions, end user feedback, or research studies? | Apps that had demonstrated being efficacious in a published clinical efficacy trial were coded as Y. |
| F |  | 6. Does the app have a clinical/recovery foundation relevant to your intended use? | Apps that described in the label a set of components consistent with best-practice smoking cessation guidelines were coded as Y. |
| G | Usability | 7. Does the app seem easy to use? | Apps that included app images that clearly suggested how the app functioned and could be used were coded as Y. |
| H | Data integration towards therapeutic goal | 8. Can data be easily shared and interpreted in a way that’s consistent with the stated purpose of the app? | Apps that either described data sharing features in the label, or that showed data sharing features in the app images and screens were coded as Y. |

Note: 1: Adapted item; Y: Yes; N: No.
